# PULPe Best Paper Award 2024

**DOI:** 10.1177/17531934251388743

**Published:** 2025-11-16

**Authors:** Tobias Laurell, Wiebke Hülsemann, Daniel Weber

**Affiliations:** 1 Department of Hand Surgery, Södersjukhuset, Stockholm, Sweden; 2 Department of Clinical Science and Education, Södersjukhuset, Karolinska Institutet, Stockholm, Sweden; 3 Department of of Hand Surgery, Catholic Children’s Hospital Wilhelmstift, Hamburg, Germany; 4 Division of Hand Surgery, Department of Paediatric Surgery, University Children’s Hospital Zurich, Zurich, Switzerland

Dear Editor,

The Paediatric Upper Limb Project Europe (PULPe) is a collaborative network of professionals across Europe dedicated to improving care for children with hand and upper limb conditions. Given the rarity and heterogeneity of conditions in this vulnerable patient group, high-quality research is both challenging and essential. The annual PULPe Best Paper Award aims to promote collaboration and recognize excellence in the field.

For the 2024 awards, we reviewed articles published in leading journals including the *Journal of Hand Surgery (European)*, *Journal of Hand Surgery (American)*, *Journal of Children’s Orthopaedics*, *Hand Surgery and Rehabilitation* and the *Journal of Hand Therapy*. Each paper was independently assessed by senior PULPe members based on novelty, methodological rigour and relevance to pediatric upper limb care.

We are pleased to announce the winners of the 2024 PULPe Best Paper Awards.

## Best congenital upper limb paper

Sletten IN, Winge MI, Hellevuo C, et al. Validity and reliability of the thumb grasp and pinch assessment for children after reconstruction of congenital hypoplastic thumbs. J Hand Surg Am. 2024, 49: 1041.e1–8.

This study addresses a gap in outcome measurement for children with congenital hypoplastic thumbs. The authors validated the Thumb Grasp and Pinch Assessment (T-GAP), a structured, video-based tool that quantifies thumb use in daily activities published in 2019. Their rigorous methodology, including inter- and intrarater reliability testing across multiple centres, demonstrated strong construct and concurrent validity. Importantly, the T-GAP captures functional details often missed by traditional strength or range-of-motion metrics. This study highlights a reliable, child-friendly tool for assessing outcomes in hypoplastic thumbs, supporting standardization in congenital hand surgery

## Best pediatric miscellaneous paper

Klein C, Borowski A, Miclo M, et al. Antibiotic treatment of hand wounds in children: contribution of a decision tree. Hand Surg Rehabil. 2024, 43:101678.

This prospective study offers a pragmatic and evidence-based approach to a common challenge in children: antibiotic use in hand injuries. The authors developed and implemented a decision tree for antibiotic prescription, grounded in national guidelines and multidisciplinary consensus. Their findings based on 238 surgically treated cases showed no significant increase in surgical site infections when antibiotics were withheld in low-risk scenarios. Impressively, unnecessary antibiotic exposure was avoided in 61% of children. This work demonstrates how structured clinical pathways can reduce overtreatment, promote antimicrobial stewardship and maintain safety in pediatric surgical care.

We commend the authors of both papers for their scientific rigour and clinical relevance. Their contributions exemplify the spirit of the PULPe initiative: to improve outcomes for children through thoughtful, collaborative research. We also thank the *Journal of Hand Surgery (European Volume)* for supporting this recognition.

